# Effects of Emotional Experience in Lexical Decision

**DOI:** 10.3389/fpsyg.2016.01157

**Published:** 2016-08-09

**Authors:** Paul D. Siakaluk, P. Ian Newcombe, Brian Duffels, Eliza Li, David M. Sidhu, Melvin J. Yap, Penny M. Pexman

**Affiliations:** ^1^Psychology, University of Northern British ColumbiaPrince George, BC, Canada; ^2^Psychology, University of CalgaryCalgary, AB, Canada; ^3^Psychology, National University of SingaporeSingapore, Singapore

**Keywords:** emotional experience, lexical decision, semantic richness, conceptual knowledge, grounded cognition, visual word recognition

## Abstract

Previous research has examined the effects of emotional experience (i.e., the ease with which words evoke emotion information) in semantic categorization (SCT), word naming, and Stroop tasks (Newcombe et al., 2012; Siakaluk et al., 2014; Moffat et al., 2015). However, to date there are no published reports on whether emotional experience influences performance in the lexical decision task (LDT). In the present study, we examined the influence of emotional experience in LDT using three different stimulus sets. In Experiment 1 we used a stimulus set used by both Kousta et al. (2009; Experiment 1) and Yap and Seow (2014) that is comprised of 40 negative, 40 positive, and 40 neutral words; in Experiment 2 we used a stimulus set comprised of 150 abstract nouns; and in Experiment 3 we used a stimulus set comprised of 373 verbs. We observed facilitatory effects of emotional experience in each of the three experiments, such that words with higher emotional experience ratings were associated with faster response latencies. These results are important because the influence of emotional experience: (a) is observed in stimulus sets comprised of different types of words, demonstrating the generalizability of the effect in LDT; (b) accounts for LDT response latency variability above and beyond the influences of valence and arousal, and is thus a robust dimension of conceptual knowledge; (c) suggests that a richer representation of emotional experience provides more reliable evidence that a stimulus is a word, which facilitates responding in LDT; and (d) is consistent with grounded cognition frameworks that propose that emotion information may be grounded in bodily experience with the world (Barsalou, 2003, 2009; Vigliocco et al., 2009).

## Introduction

Grounded cognition is the theoretical perspective that much of conceptual knowledge is derived through bodily experience with the world. Initially, the theoretical and empirical focus of cognitive scientists using grounded cognition frameworks was to examine how *sensorimotor* interactions with the world influence the acquisition and retrieval of conceptual knowledge (Meteyard and Vigliocco, 2008). According to Barsalou's (1999) perceptual symbol systems framework, conceptual knowledge is inherently multimodal, in that its different facets are stored and retrieved from neural systems dedicated to processing specific kinds of sensorimotor information. For example, knowledge of the concept “peach” would be stored in neural systems dedicated to processing visual, olfactory, gustatory, and tactile information of peaches, as well as neural systems dedicated to processing motor information, such as how one's body can interact with peaches (e.g., reaching for and picking up a peach from a bowl and then bringing it to one's mouth to bite it). Conceptual processing occurs via simulation, or the partial reenactment of the different neural states that were involved during previous bodily experience with a particular concept. For the example of “peach,” conceptual understanding of what peaches taste like arises from simulation of previous gustatory experiences involving peaches, whereas conceptual understanding of what one can physically do with peaches arises from simulation of previous motor experiences involving peaches.

The perceptual symbol systems framework has been expanded to include the notion of situated conceptualization (Barsalou, 2003, 2009; Wilson-Mendenhall et al., 2011; Barrett et al., 2014), which is the idea that situated context plays an integral role in the development of conceptual knowledge. Continuing with the example of the “peach” concept, most people have a much more nuanced conceptual understanding of peaches than merely what they look or taste like (as important as that is), and this is a result of information gained from different situational contexts in which one may have experiences with peaches. For example, part of our understanding that peaches are fruit may arise from the fact that they are sold in the produce section or that they grow on trees; situational contexts that allow for relevant bodily experiences in order to gain these types of conceptual knowledge (e.g., when walking through the grocery section of a supermarket one can see that peaches are sold in close proximity with other kinds of fruit, or when walking through an orchard one may see peach trees intermixed with apple or cherry trees).

As ubiquitous as sensorimotor experience is in our lives, emotionality is also an integral aspect of human experience. Recent theoretical work has incorporated this fact and begun to examine how emotion information may be involved in the acquisition and retrieval of conceptual knowledge (e.g., Dolan, 2002; Parisi, 2011). In addition to providing a compelling account of how situational context allows for varied sensorimotor experiences to become part of conceptual knowledge, situated conceptualization also provides an account of how situational context allows for varied *emotional* experiences to be represented. One possibility is that internal emotion systems track important environmental features and events in such a way that emotion information plays a key role in the development of conceptual knowledge. Imagine a situation in which your grandparents are visiting, and from previous experience you know that it is highly likely that they will bake your favorite kind of pie—peach pie! After a long day at work, you may be excited by the possibility of coming home to a freshly baked peach pie, and derive physical and emotional pleasure from the experience. The particular and various aspects of this experience—the presence of your grandparents, the infrequency of their visits and subsequent baking of peach pies, knowing they baked the pie for you, the pleasant conversation, and the bodily experience of eating a slice of the freshly baked peach pie—are all pertinent to your particular conceptual understanding of “peach.” Similarly, in Vigliocco et al. (2009) framework of semantic representation, emotion information is proposed to be an important aspect of conceptual knowledge, and grounded in bodily experience. They also suggested that emotion information might play more of a role for abstract concepts than concrete ones.

There has been a recent increase in research efforts to better understand how emotion information may influence the retrieval of conceptual knowledge in lexical processing. In a paper in the original *Meaning in mind: Semantic richness effects in language processing* Research Topic (Pexman et al., 2013), Newcombe et al. (2012) derived a new dimension of emotionality they called *emotional experience*, which measures the ease with which words evoke emotional experiences or information. The intuition underlying the development of this particular emotionality dimension was that concepts may vary in the extent to which they are associated with knowledge gained through emotion experience. For example, Newcombe et al. proposed that it is more likely that emotion information is relevant to the concept “justice” (e.g., there may be numerous situational contexts in which “justice” occurs, such as in courtrooms, playgrounds, and family rooms, and may be associated with a variety of emotional responses, such as joy, dismay, anger, or frustration) than to the concept “moment,” for which it is difficult to think of any kind of emotion information that would be relevant. Siakaluk et al. (2014) further suggested that emotional experience captures core person-environment regularities involved in emotionality that are present across the variety of instances in which conceptual knowledge is relevant. They used the example of the concept “crisis.” The instances in which conceptual knowledge pertinent to this concept are evoked will have many differences, such as the people, objects, or events involved. However, there are likely to be certain person-environment regularities across these different instances, such as something going terribly wrong for an individual, certain (minimal) levels of physical, psychological, and/or emotional intensity for the individuals involved in the situation, and the need for one or more individuals to react quickly and effectively, among other regularities. Thus, Siakaluk et al. proposed that the dimension of emotional experience captures the shared, core, and consistent *emotional* states that are experienced in different situated bodily contexts.

To date, several studies have examined the effects of emotional experience in semantic categorization (SCT), word naming, and Stroop tasks, but not yet in the lexical decision task (LDT). Newcombe et al. (2012) reported that emotional experience facilitated processing for abstract words in SCT (using the “Is the word abstract?” decision category) but inhibited processing for concrete words in SCT (using the “Is the word concrete?” decision category). Moffat et al. (2015) replicated the above pattern of emotional experience effects in a verbal SCT, in which their participants made verbal responses rather than button press responses. Moffat et al. further reported facilitatory emotional experience effects for abstract words, but not concrete words, in word naming. In addition, Siakaluk et al. (2014) reported that emotional experience interfered with color naming performance for abstract words in the Stroop task. Importantly, in the Moffat et al. and Siakaluk et al. studies, the effects of emotional experience were robust even after the influence of other measures of emotion information (valence and arousal) were statistically removed.

The purpose of the present study was to examine the effects of emotional experience in LDT using different types of words (i.e., words selected on a categorical basis on the dimension of valence, Experiment 1; abstract nouns, Experiment 2; and verbs, Experiment 3). We felt this was important for two primary and related reasons. First, LDT is the most frequently used task in the visual word recognition literature for examining semantic richness effects (Pexman, 2012). Second, there is a growing literature demonstrating that valence, which is the positive-negative (or pleasant-unpleasant) dimension of emotionality, and arousal, which is the calm-excited dimension of emotionality (Bradley and Lang, 1999), influence responding in LDT (e.g., Estes and Adelman, 2008; Larsen et al., 2008; Kousta et al., 2009; Adelman and Estes, 2013; Kuperman et al., 2014; Recio et al., 2014; Scott et al., 2014; Vinson et al., 2014; Yap and Seow, 2014; Imbir et al., 2016). As noted, Moffat et al. (2015) and Siakaluk et al. (2014) reported that the effects of emotional experience were robust in SCT, word naming, and Stroop tasks when the influences of valence and arousal were statistically removed. We wanted to examine whether the same would be true in LDT. We examined these issues in three LDTs; as noted, each LDT used a different stimulus set comprised of different types of words.

## Experiment 1

The goal of our first experiment was to investigate whether emotional experience might play an explanatory role in the pattern of results observed for the negative, positive, and neutral words used in two previous LDT studies: Kousta et al. (2009; Experiment 1) and Yap and Seow (2014). This particular pattern of results involved faster LDT latencies to the negative and positive words compared to the neutral words, and no difference in latencies to the negative and positive words. The reason we used this particular stimulus set is because it seemed likely that relatively high negative and high positive words should also be relatively high on emotional experience, as compared to the neutral words. To this end, emotional experience ratings were collected from 30 undergraduate students from the University of Northern British Columbia (UNBC) (the instructions for collecting emotional experience ratings are provided in the Supplementary Material). The mean valence, arousal, and emotional experience ratings for the negative, positive, and neutral words are shown in Table 1,^1^. In addition to the obvious (and intended) significant differences between the three word types on the dimension of valence, one-way between-items analyses of variance (ANOVA) revealed: (a) a significant effect of arousal, *F*_2(2, 115)_ = 21.55, *MSe* = 0.95, *p* < 0.001, $\eta_{p}^{2}$ = 0.27, with follow up tests showing that the negative words (*p*_2_ < 0.001) and the positive words (*p*_2_ < 0.001) had significantly higher arousal ratings than the neutral words, and that there was no difference in arousal ratings between the negative and positive words (*p*_2_ = 0.391); and (b) a significant effect of emotional experience, *F*_2(2, 115)_ = 28.57, *MSe* = 1.31, *p* < 0.001, $\eta_{p}^{2}$ = 0.33, with follow up tests showing that the negative words (*p*_2_ < 0.001) and the positive words (*p*_2_ < 0.001) had significantly higher emotional experience ratings than the neutral words, and that there was no difference in emotional experience ratings between the negative and positive words (*p*_2_ = 0.745). Thus, we can conclude that the negative and positive words in this stimulus set were not equivalent to the neutral words in either rated arousal or emotional experience.

**Table 1:** **Descriptive statistics for the negative, positive, and neutral words used in Experiment 1**.

**Variable**	**Negative**		**Positive**		**Neutral**	
	***M***	***SD***	***M***	***SD***	***M***	***SD***
Concreteness	437	109.6	440	103.1	462	99.6
Imageability	497	70.5	503	79.6	486	85.5
Age of acquisition	395	82.4	385	105.3	400	103.6
Familiarity	520	46.2	523	51.3	517	53.7
Log frequency	9.3	1.2	9.2	1.3	9.2	1.3
Orthographic neighborhood	3.9	6.0	4.0	5.7	3.9	5.7
Letters	5.8	1.5	5.8	1.6	5.8	1.5
Syllables	1.8	0.8	1.9	0.7	1.8	0.7
Morphemes	1.2	0.4	1.3	0.6	1.2	0.4
Mean positional bigram frequency	3259	1723	2923	1357	3103	1657
Emotional experience	4.3	1.0	4.4	1.2	2.6	1.2
Valence	2.5	0.6	7.5	0.6	5.1	0.2
Extremity of valence	2.5	0.6	2.5	0.6	0.1	0.1
Arousal	5.6	0.9	5.8	1.2	4.5	0.7

With these emotional experience ratings in hand, we turned to the main purpose of Experiment 1: to investigate whether there were effects of emotional experience above and beyond any influence of valence and arousal in LDT responses to this stimulus set used by Kousta et al. (2009; Experiment 1) and Yap and Seow (2014). The Kousta et al. study involved a yes/no procedure (in which overt responses are made to both the experimental words and the nonwords), whereas the Yap and Seow study used both a yes/no procedure and a go/no-go procedure (in which overt responses are made to only the experimental words and no responses are made to the nonwords). Because Yap and Seow reported the same general pattern of effects as Kousta et al. using both procedures, we too used the yes/no and go/no-go procedures to provide as full an examination of the influence of emotional experience as possible for this stimulus set. The hypothesis we tested in Experiment 1 was that there would be significant effects of emotional experience above and beyond any effects of valence and arousal.

### Method

#### Ethics approval and informed consent

All of the experiments reported here were conducted with the approval of the Research Ethics Boards at UNBC and the University of Calgary (UC), and all participants herein gave their written informed consent to participate at their institution of study.

#### Participants

Two groups of UNBC undergraduate students participated in Experiment 1: one group of 34 individuals participated in the yes/no LDT and a different group of 33 individuals participated in the go/no-go LDT. All participants were native English speakers, reported normal or corrected-to-normal vision, and received bonus course credit for their participation.

#### Stimuli

Kousta et al. (2009) collected valence and arousal ratings for a large set of words using the procedures in ANEW (Bradley and Lang, 1999), and merged their ratings with those of the ANEW database. Based on these ratings, they selected 40 negative words, 40 positive words, and 40 neutral words. These words served as the stimuli for their Experiment 1, as well as for the two experiments reported by Yap and Seow (2014). We also used these words in the present experiment. These stimuli were matched for the following characteristics: concreteness, imageability, and familiarity (all from the MRC psycholinguistic database; Coltheart, 1981), log frequency, orthographic neighborhood, number of letters, syllables, and morphemes, and mean positional bigram frequency (all from the English Lexicon Project database; Balota et al., 2007), and age of acquisition (from the Bristol norms database; Stadthagen-Gonzalez and Davis, 2006). As noted, the emotional experience ratings were collected from 30 UNBC undergraduate students (these individuals did not participate in any of the subsequent experiments described below). Means and *SD*s for the predictor variables are shown in Table 1. The 120 nonwords used by Yap and Seow (2014) were used in the present experiment and were matched to the words on number of letters.

#### Apparatus and procedure

The 120 words and 120 nonwords were presented in both LDTs. For the yes/no LDT, participants were instructed to respond to the words by pressing the “?” key and to the nonwords by pressing the “z” key on the computer keyboard. For the go/no-go LDT, participants were instructed to respond only to the words, again by pressing the “?” key. They were further instructed not to respond to the nonwords, and were told that if no response was made the stimulus would be automatically replaced by the next stimulus after 2500 ms. All participants were instructed to make their responses as quickly and as accurately as possible. The stimuli were presented in the center of a color VGA monitor driven by a Pentium-class microcomputer running DirectRT software (http://www.empirisoft.com/DirectRT.aspx). A trial was initiated by a fixation marker that appeared at the center of the computer display for 1000 ms and was then replaced by a stimulus item. The inter-trial interval was 2000 ms. Stimulus order was randomized separately for each participant. Following every 60 trials, participants had an opportunity to take a break, and continued when ready by pressing the spacebar. Before beginning either LDT, participants were given practice trials consisting of 10 words and 10 nonwords.

#### Data analysis

In the first analysis for each LDT we used ANOVA to determine if our results replicated the main effect of valence observed in Kousta et al.'s (2009; Experiment 1) yes/no experiment and in Yap and Seow's (2014) yes/no and go/no-go experiments. Both by-subjects (*F*_1_) and by-items (*F*_2_) ANOVAs were conducted. In the second analysis for each LDT we used by-subjects (*F*_1_) ANOVA to determine if our results replicated the effects of valence in response latency distributional analyses in order to provide a full attempt at replicating the results of Yap and Seow. Lastly, we conducted separate hierarchical multiple regression analyses for the yes/no and go/no-go LDTs. We followed the coding of valence scheme used by Adelman and Estes (2013): valence ratings and extremity of valence values (i.e., the absolute distance from the midpoint of the valence scale) were entered together. Adelman and Estes proposed that the extremity of valence dimension determines whether both positive and negative stimuli are responded to differently than neutral stimuli, whereas the valence dimension determines whether positive stimuli are responded to differently than negative stimuli. As such, this coding scheme provides a thorough test of potential effects of the valence dimension. This valence coding scheme was used in all three experiments reported in the present study. In step 1 of the regression analyses we entered the following predictors: concreteness, imageability, familiarity, log frequency, orthographic neighborhood, number of letters, syllables, and morphemes, mean positional bigram frequency, and age of acquisition. These step 1 variables have all been shown to influence lexical processing, and we wanted to account for variance due to these non-emotion factors before the emotion dimensions were entered, in order to evaluate the effects of each emotion dimension independent of these factors. In step 2 we entered the following predictors: valence, extremity of valence, and arousal. In step 3 we entered emotional experience.

### Results and discussion

The following procedure for removal of outliers was used for each LDT. First, response latencies faster than 250 ms or slower than 2000 ms were treated as outliers and removed from the data sets. Second, for each participant, response latencies greater than 2.5 *SD*s from the cell mean of each condition were treated as outliers and removed from the data sets. A total of 117 responses (2.89% of the data) and 144 responses (3.70% of the data) were removed by this procedure from the yes/no and go/no-go LDTs, respectively. Word response errors comprised only 2.12 and 0.67% of trials from the yes/no and go/no-go LDTs, respectively. As such, the response error data were not analyzed. The raw lexical decision latencies were *z* score transformed for the multiple regression analyses. Zero-order correlations between the predictor variables and the criterion variables are presented in Table 2.

**Table 2:** **Zero-order correlations between the criterion variables and the predictor variables in Experiment 1**.

**Measure**	**1**	**2**	**3**	**4**	**5**	**6**	**7**	**8**	**9**	**10**	**11**	**12**	**13**	**14**	**15**	**16**
1. Concreteness	−															
2. Imageability	0.79^*^	−														
3. AoA	−0.18^*^	−0.36^*^	−													
4. Familiarity	−0.11	0.02	−0.52^*^	−												
5. Log frequency	−0.32^*^	−0.29^*^	−0.27^*^	0.56^*^	−											
6. N	0.07	0.13	−0.37^*^	0.18^*^	0.25^*^	−										
7. Letters	−0.12	−0.13	0.25^*^	0.01	−0.09	−0.72^*^	−									
8. Syllables	−0.16	−0.14	0.21^*^	0.03	−0.01	−0.54^*^	0.72^*^	−								
9. Morphemes	−0.31^*^	−0.19^*^	0.25^*^	0.06	0.05	−0.26^*^	0.42^*^	0.53^*^	−							
10. MPBF	−0.00	−0.00	0.09	0.08	−0.02	−0.36^*^	0.65^*^	0.38^*^	0.20^*^	−						
11. EE	−0.42^*^	−0.13	−0.04	0.28^*^	0.27^*^	−0.06	0.21^*^	0.17	0.30^*^	0.14	−					
12. Valence	−0.01	0.03	−0.07	0.08	0.03	0.01	0.03	0.04	0.09	−0.08	0.04	−				
13. Extremity	−0.09	0.13	−0.02	0.04	−0.00	−0.02	−0.00	0.00	0.06	0.00	0.63^*^	0.01	−			
14. Arousal	−0.12	0.06	−0.02	0.03	0.08	0.07	−0.00	0.03	0.09	−0.03	0.55^*^	0.09	0.53^*^	−		
15. Yes/no	0.20^*^	0.03	0.30^*^	−0.48^*^	−0.52^*^	−0.14	0.11	0.05	−0.07	0.15	−0.61^*^	−0.15	−0.45^*^	−0.34^*^	−	
16. Go/no-go	0.18	0.01	0.36^*^	−0.53^*^	−0.51^*^	−0.12	0.09	0.02	−0.05	0.10	−0.56^*^	−0.06	−0.33^*^	−0.22^*^	0.77^*^	−

* *p < 0.05. AoA, age of acquisition; N, orthographic neighborhood; MPBF, mean positional bigram frequency; EE, emotional experience; Yes/no, yes/no lexical decision task latency; Go/no, go lexical decision task latency*.

#### Yes/no LDT

The ANOVA results included a main effect of valence, *F*_1(2, 66)_ = 47.31, *MSe* = 376.74, *p* < 0.001, $\eta_{p}^{2}$ = 0.59; *F*_2(2, 113)_ = 13.05, *MSe* = 1713.15, *p* < 0.001, $\eta_{p}^{2}$ = 0.19. As was the case with the Kousta et al. (2009) and Yap and Seow (2014) studies, both the negative words (*M* = 649, *SD* = 36.9) (*p*_1_ < 0.001; *p*_2_ < 0.001) and the positive words (*M* = 636, *SD* = 41.0) (*p*_1_ < 0.001; *p*_2_ < 0.001) were responded to faster than the neutral words (*M* = 683, *SD* = 46.3). However, unlike the above studies, here the positive words were responded to faster than the negative words, although the effect was significant only in the subjects analysis (*p*_1_ = 0.003) and not in the items analysis (*p*_2_ = 0.151).

As noted, Yap and Seow also conducted response latency distributional analyses, and for completeness of replication of their study, we too conducted response latency distributional analyses. In order to do so, ex-Gaussian parameters, (μ, σ, τ) were obtained using the quantile maximum likelihood estimation procedure in Cousineau et al. (2004) QMPE program. Distributional shifts are reflected by changes in μ, whereas modulations in the tail of the distribution are reflected by τ. This allows changes in mean response latency to be partitioned into distributional shifting (μ) and distributional skewing (τ). Raw data were fitted to the theoretical ex-Gaussian function using the maximum number of quantiles, and all fits converged within 250 iterations. For μ, there was a main effect of valence, *F*_1(2, 66)_ = 10.54, *MSe* = 848.20, *p* < 0.001, $\eta_{p}^{2}$ = 0.24; the negative words (*M* = 541, *SD* = 70.4) (*p* = 0.005) and the positive words (*M* = 535, *SD* = 62.6) (*p* < 0.001) were responded to faster than the neutral words (*M* = 566, *SD* = 79), but there was no significant difference between the positive and negative words. For σ and τ, the main effect of valence was not significant.

The results of the regression analysis are shown in the top half of Table 3,^2^. In step 2, there was a significant change in latency variability (Δ*R*^2^ = 0.16). The only predictor in step 2 that accounted for a significant amount of unique variability was extremity of valence (*sr* = −0.29), which indicates that faster latencies were associated with more extreme valence values, regardless of valence polarity. In step 3, there was a significant change in latency variability (Δ*R*^2^ = 0.08), with faster latencies associated with higher emotional experience ratings (*sr* = −0.28). Thus, in the yes/no LDT, our hypothesis was supported, such that the dimension of emotional experience accounted for unique latency variability above and beyond that accounted for by valence, extremity of valence, and arousal.

**Table 3:** **Results of hierarchical multiple regression analyses of response latencies for Experiment 1**.

**Variable**	***B***	***SEB***	**β**	***sr***	***ΔR*^2^**	***R*^2^**
**YES/NO LDT**						
Step 1 (control variables)						0.41^***^
Concreteness	0.00	0.00	0.35	0.20^**^		
Imageability	−0.00	0.00	−0.35	−0.19^*^		
AoA	0.00	0.00	0.06	0.04		
Familiarity	−0.00	0.00	−0.28	−0.20^**^		
Log frequency	−0.08	0.02	−0.38	−0.29^***^		
N	0.01	0.01	0.18	0.11		
Letters	0.03	0.02	0.15	0.08		
Syllables	0.02	0.04	0.04	0.03		
Morphemes	−0.04	0.06	−0.06	−0.05		
MPBF	0.00	0.00	0.12	0.11		
Step 2					0.16^***^	0.57^***^
Valence	−0.01	0.01	−0.07	−0.07		
Extremity	−0.08	0.02	−0.36	−0.29^***^		
Arousal	−0.03	0.02	−0.11	−0.09		
Step 3					0.08^***^	0.65^***^
Emotional experience	−0.09	0.02	−0.48	−0.28^***^		
**GO/NO-GO LDT**						
Step 1 (control variables)						0.40^***^
Concreteness	0.00	0.00	0.28	0.16^*^		
Imageability	−0.00	0.00	−0.25	−0.14^†^		
AoA	0.00	0.00	0.15	0.11		
Familiarity	−0.00	0.00	−0.29	−0.21^**^		
Log frequency	−0.08	0.03	−0.32	−0.24^**^		
N	0.01	0.01	0.18	0.12		
Letters	0.03	0.03	0.13	0.07		
Syllables	0.03	0.05	0.06	0.04		
Morphemes	−0.04	0.07	−0.06	−0.05		
MPBF	0.00	0.00	0.02	0.02		
Step 2					0.08^**^	0.48^***^
Valence	−0.00	0.01	−0.01	−0.01		
Extremity	−0.07	0.02	−0.26	−0.21^**^		
Arousal	−0.02	0.03	−0.07	−0.06		
Step 3					0.10^***^	0.58^***^
Emotional experience	−0.13	0.03	−0.55	−0.32^***^		

† p = 0.071;

* *p < 0.05*,

** *p < 0.01*,

*** *p < 0.001. AoA, age of acquisition; N, orthographic neighborhood; MPBF, mean positional bigram frequency*.

#### Go/no-go LDT

The sphericity assumption was violated in the ANOVA by subjects, so we used the Greenhouse-Geisser correction. There was a main effect of valence, *F*_1(1.550, 48.961)_ = 11.89, *MSe* = 1753.79, *p* < 0.001, $\eta_{p}^{2}$ = 0.27; *F*_2(2, 113)_ = 6.70, *MSe* = 2766.75, *p* = 0.002, $\eta_{p}^{2}$ = 0.11. As was the case with the Yap and Seow (2014) go/no-go LDT, both the negative words (*M* = 627, *SD* = 47.5) (*p*_1_ = 0.001; *p*_2_ = 0.003) and the positive words (*M* = 623, *SD* = 43.8) (*p*_1_ = 0.001; *p*_2_ = 0.001) were responded to faster than the neutral words (*M* = 664, *SD* = 65.5). Also consistent with the Yap and Seow results, there was no difference between latencies for the negative and positive words (*p*_1_ = 0.633; *p*_2_ = 0.718).

In the response latency distributional analysis, for μ, there was a main effect of valence, *F*_1(2, 64)_ = 4.43, *MSe* = 625.51, *p* = 0.016, $\eta_{p}^{2}$ = 0.12; the negative words (*M* = 513, *SD* = 45.6) (*p* = 0.039) and the positive words (*M* = 507, *SD* = 50.4) (*p* = 0.015) were responded to faster than the neutral words (*M* = 525, *SD* = 50.6), but there was no significant difference between the positive and negative words. For σ and τ, the main effect of valence was not significant.

The results of the response latency distributional analyses for the yes/no and go/no-go LDTs of the present experiment are partly consistent with Yap and Seow's (2014) findings. Like Yap and Seow, there were valence effects in the pattern in mean response latencies, as indicated by μ, such that responses to the positive and negative words were faster than to the neutral words, but there was no difference between the positive and negative words. Interestingly, unlike Yap and Seow, there were no valence effects mediated by τ in either LDT. Although the reasons for this discrepancy are unclear, the results suggest that valence effects in the present experiment predominately reflect earlier lexical processes rather than later processes that are specific to word/nonword discrimination.

The results of the regression analysis are shown in the bottom half of Table 3. In step 2, there was a significant change in latency variability (Δ*R*^2^ = 0.08). Again, the only predictor in step 2 that accounted for a significant amount of unique variability was extremity of valence (*sr* = −0.21). In step 3, there was a significant change in latency variability (Δ*R*^2^ = 0.10), with faster latencies associated with higher emotional experience ratings (*sr* = −0.32). As was the case in the yes/no LDT, our hypothesis was supported in the go/no-go LDT, such that emotional experience accounted for a significant amount of unique latency variability above and beyond that accounted for by valence, extremity of valence, and arousal.

Using the experimental stimuli of Kousta et al. (2009; Experiment 1) and Yap and Seow (2014), we replicated their findings of faster latencies to negative and positive words than to neutral words in both yes/no and go/no-go LDTs. For both LDTs, in step 2 only extremity of valence accounted for a significant amount of unique latency variability. Latencies were faster to the negative and positive words than to the neutral words, but there were no differences in latencies between the negative and positive words (recall that the slight latency advantage to the positive words in the yes/no LDT did not reach statistical significance in the items analysis). However, the key and novel finding from Experiment 1, as revealed in the regression analyses, was that emotional experience influenced LDT performance above and beyond any influence of valence, extremity of valence, and arousal. These facilitatory effects of emotional experience support the idea that this dimension of emotion information is partly driving the reported valence effects by Kousta et al. (2009; Experiment 1) and Yap and Seow (2014), and is thus an integral component of lexical-semantic knowledge, independent of the pleasant-unpleasant or calm-excited dimensions underlying lexical-semantic knowledge. We will further consider ways in which the dimension of emotional experience might differ from those of valence and arousal in the General Discussion. This is the first reported instance of facilitatory effects of emotional experience in LDT.

## Experiment 2

One could argue that perhaps the emotional experience effects observed in Experiment 1 were a result of the categorical manner in which the stimuli were originally selected; that is, because the items were specifically chosen for their negative, positive, or neutral meanings. Since two-thirds of the stimuli used in Experiment 1 were very negative or very positive, the impact of emotional experience might have been an artifact of the way these stimuli were selected. To address this issue, and to examine whether these results would also be observed in LDT using a different stimulus set, we conducted Experiment 2. In Experiment 2 we used a different stimulus set that was: (a) not chosen on a categorical basis on the valence dimension (or on the dimensions of emotional experience or arousal); and (b) relatively more abstract, in order to test the generality of emotional experience effects in LDT, because the words used in Experiment 1 were relatively more concrete and easily imageable (compare the mean concreteness and imageability ratings for the stimuli used in Experiment 1, as shown in Table 1, with those used in Experiment 2, as shown in Table 4).

**Table 4:** **Descriptive statistics for the abstract nouns used in Experiment 2**.

**Variable**	**Abstract words**	
	***M***	***SD***
Concreteness	2.6	0.7
Imageability	3.0	0.6
Age of acquisition	9.2	2.1
Familiarity	517.1	79.3
Log frequency	9.3	1.5
Orthographic neighborhood	1.4	3.1
Letters	7.0	1.6
Syllables	2.3	0.7
Morphemes	1.6	0.6
Mean positional bigram frequency	3836.6	1867.4
Emotional experience	3.5	1.3
Valence	5.3	1.3
Extremity of valence	1.1	0.8
Arousal	4.3	0.8

### Method

#### Participants

Fifty UNBC undergraduate students participated in the experiment; none of these individuals participated in Experiment 1. All participants were native English speakers, reported normal or corrected-to-normal vision, and received bonus course credit for their participation.

#### Stimuli

One hundred-fifty abstract nouns were used in the present experiment. These stimuli were selected from the Toronto Word Pool (Friendly et al., 1982), and the Paivio et al. (1968) work banks and had concreteness and imageability ratings of 4.0 or less. As was the case for Experiment 1, in addition to norms for concreteness and imageability, we obtained norms for familiarity (for 126 stimuli from the MRC psycholinguistic database, and we collected familiarity norms for the remaining 24 stimuli from a different group of 26 UNBC undergraduate students using the same instructions as used by Toglia and Battig, 1978), log frequency, orthographic neighborhood, number of letters, syllables, and morphemes, and mean positional bigram frequency (all from the English Lexicon Project database), and age of acquisition (from the Bristol norms database). The emotional experience ratings were collected by Newcombe et al. (2012), using the same instructions as provided in the Supplementary Material. Means and *SD*s for the predictor variables are shown in Table 4,^3^. One-hundred fifty nonwords, matched to the words on number of letters, were used in the present experiment.

#### Apparatus, procedure, and data analysis

The apparatus, procedure, and hierarchical multiple regression analysis used in the present experiment were identical to those used for the go/no-go LDT of Experiment 1.

### Results and discussion

The procedure used for the removal of outliers was the same as that used in Experiment 1. This procedure resulted in removal of a total of 272 responses (3.63% of the data) from the data set. Word response errors comprised only 1.43% of trials, and as such, the response error data were not analyzed. The raw lexical decision latencies were *z* score transformed. Zero-order correlations between the predictor variables and the criterion variable are presented in Table 5.

**Table 5:** **Zero-order correlations between the criterion variable and the predictor variables in Experiment 2**.

**Measure**	**1**	**2**	**3**	**4**	**5**	**6**	**7**	**8**	**9**	**10**	**11**	**12**	**13**	**14**	**15**
1. Concreteness	−														
2. Imageability	0.06	−													
3. AoA	−0.04	−0.30^*^	−												
4. Familiarity	0.27^*^	0.07	−0.60^*^	−											
5. Log frequency	0.18^*^	0.01	−0.52^*^	0.61^*^	−										
6. N	−0.09	0.01	−0.24^*^	0.12	0.12	−									
7. Letters	−0.19^*^	0.05	0.33^*^	−0.23^*^	−0.33^*^	−0.45^*^	−								
8. Syllables	−0.22^*^	−0.04	0.39^*^	−0.29^*^	−0.32^*^	−0.38^*^	0.71^*^	−							
9. Morphemes	−0.15	−0.11	0.29^*^	−0.17^*^	−0.23^*^	−0.20^*^	0.63^*^	0.56^*^	−						
10. MPBF	0.06	0.09	0.02	0.05	−0.09	−0.09	0.48^*^	0.16^*^	0.21^*^	−					
11. EE	−0.35^*^	0.50^*^	−0.23^*^	−0.06	−0.04	0.07	−0.01	−0.06	−0.13	0.03	−				
12. Valence	−0.17^*^	−0.08	−0.17^*^	0.12	0.30^*^	−0.00	−0.08	0.03	−0.14	−0.08	−0.01	−			
13. Extremity	−0.15	0.39^*^	−0.26^*^	0.02	0.09	−0.01	−0.06	−0.15	−0.19^*^	−0.01	0.66^*^	−0.07	−		
14. Arousal	−0.13	0.41^*^	−0.12	−0.15	−0.10	0.09	0.05	−0.03	−0.08	0.02	0.57^*^	−0.07	0.44^*^	−	
15. Latencies	−0.13	−0.08	0.62^*^	−0.62^*^	−0.63^*^	−0.15	0.35^*^	0.32^*^	0.27^*^	0.07	−0.18^*^	−0.21^*^	−0.01	−0.15	−

* *p < 0.05. AoA, age of acquisition; N, orthographic neighborhood; MPBF, mean positional bigram frequency; EE, emotional experience*.

The hierarchical multiple regression results are shown in Table 6. In step 2, there was no significant change in latency variability (Δ*R*^2^ = 0.01) when valence, extremity of valence, and arousal were added to the analysis. In interpreting this finding it is important to note that the ranges of valence and arousal ratings were not very large (see Table 4). In step 3, there was a significant change in latency variability (Δ*R*^2^ = 0.03): faster latencies were associated with higher emotional experience ratings (*sr* = −0.18). Thus, using a new set of stimuli that consisted of abstract nouns, we again observed a significant facilitatory effect of emotional experience in LDT, above and beyond any influence of valence, extremity of valence, and arousal. Thus, the results of Experiment 2 suggest that the facilitatory effects of emotional experience observed in Experiment 1 were not simply due to the categorical manner in which the stimuli were selected (i.e., being specifically chosen because they varied on the valence dimension), and that such effects are observed for relatively more abstract nouns in LDT.

**Table 6:** **Results of hierarchical multiple regression analysis of response latencies in Experiment 2**.

**Variable**	***B***	***SEB***	**β**	***sr***	***ΔR*^2^**	***R*^2^**
Step 1 (control variables)						0.56^***^
Concreteness	0.01	0.03	0.03	0.02		
Imageability	0.01	0.04	0.01	0.01		
AoA	0.05	0.01	0.29	0.20^**^		
Familiarity	−0.00	0.00	−0.27	−0.19^**^		
Log frequency	−0.06	0.02	−0.27	−0.21^**^		
N	0.01	0.01	0.04	0.04		
Letters	0.04	0.03	0.17	0.09		
Syllables	−0.04	0.04	−0.08	−0.05		
Morphemes	0.02	0.04	0.04	0.03		
MPBF	−0.00	0.00	−0.02	−0.01		
Step 2					0.01	0.57^***^
Valence	−0.01	0.02	−0.02	−0.02		
Extremity	−0.01	0.03	−0.03	−0.02		
Arousal	−0.03	0.03	−0.06	−0.05		
Step 3					0.03^**^	0.60^***^
Emotional experience	−0.08	0.03	−0.31	−0.18^**^		

** *p < 0.01*,

*** *p < 0.001. AoA, age of acquisition; N, orthographic neighborhood; MPBF, mean positional bigram frequency*.

## Experiment 3

The stimulus set used in Experiment 1 consisted of words selected on a categorical basis along the dimension of valence and in Experiment 2 consisted of nouns that were relatively abstract. In contrast, for Experiment 3 we selected a stimulus set that consisted entirely of verbs. The semantic richness literature has tended to focus on noun stimuli; verbs are a class of words that has not been as extensively examined for semantic richness effects (cf. Sidhu et al., 2014, 2016). This is especially the case for the investigation of the influence of dimensions of emotion information. Thus, the purpose of Experiment 3 was to examine whether emotional experience influences the processing of verbs in LDT above and beyond any influence of valence, extremity of valence, and arousal. If so, then this finding would suggest that emotional experience is an integral component of meaning for two important word classes, nouns and verbs.

### Method

The LDT data obtained for Experiment 3 were from Sidhu et al. (2014; Experiment 1). In that study, 30 UC undergraduate participants made LDT responses to 400 verbs and 400 nonwords. Each trial began with an asterisk, which then was replaced by a letter string after 1500 ms. Verbs were presented in their infinitive form (e.g., *to leap*), and nonwords were also preceded by the word *to*. The raw lexical decision latencies were *z* score transformed. Sidhu et al. (2014) analyzed LDT latencies only for correct trials (the error rate was 3.45%, and as such an analysis of errors was not carried out), and excluded trials on which latencies were more than 2.5 *SD* from a participant's mean (< 1% of the data). In addition, eight verbs were excluded from the analyses due to high error rates (>30%), leaving a total of 392 verbs. These stimuli were the starting point for the present analysis.

Sidhu et al. (2014) obtained norms for concreteness (Brysbaert et al., 2014), imageability (Chiarello et al., 1999; Bird et al., 2001), age of acquisition (Kuperman et al., 2012), log frequency, orthographic neighborhood, number of letters, syllables, and morphemes, and mean positional bigram frequency (all from the English Lexicon Project database). For the purposes of the present experiment, we collected emotional experience ratings for 686 verbs (of which the 400 verbs for which Sidhu et al., 2014, collected LDT latencies were a subset). These 686 verbs were randomly assigned to one of two lists, and two groups of UC undergraduate students (33 for the first list and 34 for the second list; none of these participants provided LDT latencies that were analyzed in the present experiment) provided the emotional experience ratings using the instructions listed in the Supplementary Material. We also obtained valence and arousal norms from Warriner et al. (2013); however, there were no values for these two variables for 19 stimuli. Further, for a large number of stimuli there were no familiarity values (from the MRC psycholinguistic database), so this predictor was not included in the present experiment. As such, the regression analyses below include LDT latencies to 373 verbs. Descriptive characteristics for this subset of the Sidhu et al. (2014) stimuli are presented in Table 7. The complete set of emotional experience ratings for 686 verbs are available from http://psychology.ucalgary.ca/languageprocessing/node/22.

**Table 7:** **Descriptive statistics for the verbs used in Experiment 3**.

**Variable**	**Verbs**	
	***M***	***SD***
Concreteness	3.1	0.8
Imageability	425.0	99.6
Age of acquisition	7.8	2.4
Log frequency	8.6	1.8
Orthographic neighborhood	4.7	5.8
Letters	5.6	1.6
Syllables	1.7	0.7
Morphemes	1.3	0.5
Mean positional bigram frequency	3585	1578
Emotional experience	3.7	0.8
Valence	5.1	1.3
Extremity of valence	1.1	0.8
Arousal	4.3	0.9

### Results and discussion

Zero-order correlations between the predictor variables and the criterion variable are presented in Table 8. The hierarchical multiple regression results are shown in Table 9. In step 2, there was a significant change in latency variability (Δ*R*^2^ = 0.02). Both valence (*sr* = −0.08) and arousal (*sr* = −0.10) accounted for a significant amount of unique variability: faster latencies were associated with higher valence and arousal ratings. Due to the way Warriner et al. (2013) reverse scored (*post-hoc*) their valence and arousal ratings, these findings reveal that faster latencies were associated with words that were rated as relatively more positive and more arousing. In step 3, there was a significant change in latency variability (Δ*R*^2^ = 0.01): faster latencies were associated with higher emotional experience ratings (*sr* = −0.10). Thus, when analyzing responses to a third set of stimuli that consisted of verbs, we again observed a significant facilitatory effect of emotional experience, and this effect was above and beyond any influence of valence, extremity of valence, and arousal. The results from the present experiment demonstrate that various forms of emotion information contribute to the conceptual understanding of a class of words that includes concepts referring to actions, states, or relations; namely, verbs.

**Table 8:** **Zero-order correlations between the criterion variable and the predictor variables in Experiment 3**.

**Measure**	**1**	**2**	**3**	**4**	**5**	**6**	**7**	**8**	**9**	**10**	**11**	**12**	**13**	**14**
1. Concreteness	−													
2. Imageability	0.77^*^	−												
3. AoA	−0.51^*^	−0.51^*^	−											
4. Log frequency	−0.06	−0.10	−0.52^*^	−										
5. N	0.31^*^	0.22^*^	−0.49^*^	0.35^*^	−									
6. Letters	−0.34^*^	−0.24^*^	0.46^*^	−0.29^*^	−0.68^*^	−								
7. Syllables	−0.48^*^	−0.38^*^	0.53^*^	−0.21^*^	−0.60^*^	0.80^*^	−							
8. Morphemes	−0.26^*^	−0.27^*^	0.41^*^	−0.25^*^	−0.34^*^	0.46^*^	0.55^*^	−						
9. MPBF	−0.15^*^	−0.18^*^	0.22^*^	−0.05	−0.12^*^	0.29^*^	0.26^*^	0.15^*^	−					
10. EE	−0.26^*^	−0.02	0.07	−0.02	−0.11^*^	0.14^*^	0.13^*^	−0.07	−0.00	−				
11. Valence	−0.03	−0.02	−0.20^*^	0.22^*^	0.07	−0.01	−0.03	0.02	0.02	−0.09	−			
12. Extremity	0.16^*^	0.28^*^	−0.18^*^	0.00	0.05	0.01	−0.07	−0.20^*^	−0.04	0.49^*^	−0.12^*^	−		
13. Arousal	0.16^*^	0.27^*^	−0.04	−0.09	0.02	−0.04	−0.06	−0.09	−0.07	0.37^*^	−0.34^*^	0.38^*^	−	
14. Latencies	−0.16^*^	−0.17^*^	0.54^*^	−0.61^*^	−0.24^*^	0.33^*^	0.30^*^	0.17^*^	0.18^*^	−0.04	−0.21^*^	−0.03	−0.05	−

* *p < 0.05. AoA, age of acquisition; N, orthographic neighborhood; EE, emotional experience*.

**Table 9:** **Results of hierarchical multiple regression analyses of response latencies in Experiment 3**.

**Variable**	***B***	***SEB***	**β**	***sr***	***ΔR*^2^**	***R*^2^**
Step 1 (control variables)						0.48^***^
Concreteness	0.01	0.02	0.02	0.01		
Imageability	0.00	0.00	−0.09	−0.06		
AoA	0.04	0.01	0.27	0.16^***^		
Log frequency	−0.09	0.01	−0.51	−0.37^***^		
N	0.01	0.00	0.21	0.14^***^		
Letters	0.03	0.01	0.16	0.08^*^		
Syllables	0.04	0.03	0.08	0.04		
Morphemes	−0.09	0.03	−0.14	−0.12^**^		
MPBF	0.00	0.00	0.06	0.06		
Step 2					0.02^*^	0.50^***^
Valence	−0.02	0.01	−0.09	−0.08^*^		
Extremity	0.02	0.02	0.05	0.04		
Arousal	−0.04	0.02	−0.12	−0.10^**^		
Step 3					0.01^**^	0.51^***^
Emotional experience	−0.05	0.02	−0.13	−0.10^**^		

* *p < 0.05*,

** *p < 0.01*,

*** *p < 0.001. AoA, age of acquisition; N, orthographic neighborhood; MPBF, mean positional bigram frequency*.

## General discussion

The contributions to the second edition of the *Meaning in mind: Semantic richness effects in language processing* Research Topic examine the influence of different forms of semantic richness on various aspects of language processing. The contribution of the present study to this endeavor, as noted, was to examine whether effects of emotional experience would be observed for different types of words in LDT above and beyond any effects of valence and arousal. Previous research has demonstrated that emotional experience influences responding in SCT, word naming, and Stroop tasks (Newcombe et al., 2012; Siakaluk et al., 2014; Moffat et al., 2015). However, to date there are no published reports examining the influence of emotional experience in LDT, yet LDT is the most often used task to examine semantic richness effects in the visual word recognition literature (Pexman, 2012).

To accomplish our objective, we used three different stimulus sets: one set of words that was constructed on a categorical basis along the dimension of valence (i.e., the stimulus set consisted of negative, positive, and neutral words) (see Kousta et al., 2009; Experiment 1, and Yap and Seow, 2014), a second set consisting of abstract nouns, and a third set consisting of verbs. For each experiment, we used hierarchical multiple regression analyses. In step 1, we included a common set of control predictor variables known to account for LDT latency variability. In step 2, we included the emotionality dimensions of valence, extremity of valence, and arousal. As noted, the valence dimension allows for inferences about whether faster responses are made to more negative or to more positive words, whereas the extremity of valence dimension allows for inferences about whether faster responses are made to more negative and more positive words, regardless of valence polarity, than to neutral words (Adelman and Estes, 2013). As such, this valence coding scheme provided a thorough test of potential effects of the valence dimension. We then entered emotional experience in step 3 of our analyses. These regression analyses provided a rigorous test of the effects of emotional experience for different types of words in LDT, because if any such effects were observed, they were above and beyond any effects of the control and emotion predictor variables entered in the previous two steps of the analyses. For each experiment in the present study, we observed that emotional experience accounted for a significant amount of unique LDT latency variability, and that these effects were facilitatory, such that faster LDT latencies were associated with higher emotional experience ratings. These results indicate that effects of emotional experience in LDT generalize to stimulus sets constructed in different ways (i.e., categorized by valence, such as in Experiment 1, or not categorized on any dimension of emotionality, as in Experiments 2 and 3), and comprised of different types of words (e.g., relatively concrete words, relatively abstract nouns, and verbs).

Several inferences can be drawn from the facilitatory effects of emotional experience in the present study. First, these effects are consistent with task demands associated with the LDT. In LDT, participants are required to determine if presented stimuli are words or not. Any information, including semantic information, that provides evidence that a stimulus is a word ought to make the decision more efficient, resulting in faster and/or more accurate responding. The results of the present study demonstrate that emotional experience, which assesses the ease with which words evoke emotional experiences or information, is a dimension of emotion knowledge, above and beyond those of valence and arousal, that makes lexical decisions more efficient, and thus is an integral component of lexical semantics. Below, we offer a proposal for how emotional experience might tap into different aspects of emotion knowledge that arise from situated bodily contexts as compared to valence and arousal.

Second, facilitatory effects of emotional experience in LDT can be accounted for through the process of semantic feedback. According to the semantic feedback activation framework (Hino and Lupker, 1996), the visual word recognition system is composed of three distinct yet interconnected sets of units; namely, orthographic units (that process spelling information), phonological units (that process sound information), and semantic units (that process meaning information). Importantly, processing among one set of units may influence processing in the other sets of units via feedforward and feedback activation. Of relevance to performance in the LDT, word/nonword decisions are ultimately based on activation among orthographic units; however, activation from semantic units may feedback to orthographic units, subsequently enhancing their processing efficiency. The idea is that semantically richer words should elicit greater activation among semantic units, leading to greater feedback from these units to orthographic units, resulting in faster settling of orthographic representations, and hence faster LDT responses. This account is consistent with the pattern of effects we observed in the present study: words with higher ratings of emotional experience, which we assume have richer emotional semantic representations, were associated with faster LDT latencies.

Third, the facilitatory effects of emotional experience are consistent with different frameworks of grounded cognition. For example, according to the perceptual symbol systems framework (Barsalou, 1999) and the notion of situated conceptualization (Barsalou, 2003, 2009; Wilson-Mendenhall et al., 2011; Barrett et al., 2014), the development of conceptual knowledge is highly contingent on (or grounded in) sensory, motor, and emotion experiences across a variety of situated contexts. The semantic representation framework of Vigliocco et al. (2009) is similar in that it proposes that different types of bodily experience contribute to the development of conceptual knowledge. An important component of this latter framework is the emphasis it places on how emotion information, which is associated with internal, bodily processes that track environmental context, largely underlies the comprehension of abstract concepts.

Kiverstein and Miller (2015) have also recently emphasized that situated context and emotion knowledge play important roles in the development, processing, and retrieval of conceptual knowledge. Further, we assume that emotion knowledge is multidimensional. Thus, it is highly unlikely that just one or two dimensions of emotionality, such as valence and arousal, will be sufficient to capture the *entire* richness and complexity of knowledge gained through bodily emotion experience. In what follows, we will attempt to provide a proposal in which the contribution of emotional experience is disentangled from the contributions of valence and arousal in the development, processing, and retrieval of conceptual knowledge. To do so, we will use as examples three words that were presented as stimuli in Experiment 1.

First, take the concept “failure.” As the notion of situated conceptualization emphasizes, “failure” does not occur in a contextual vacuum, nor is it tied to just one specific situated context (Barsalou, 2003), but arises from an essentially unlimited number of situated contexts (such as sporting events, academic efforts, business endeavors, personal relationships, etc.). Because of the rich diversity of lived, bodily experience, emotion knowledge will be integral to the understanding of “failure.” In addition, as noted, emotion knowledge will be multidimensional. The two traditional dimensions of emotionality, valence and arousal, will account for important aspects of “failure.” Most obviously, “failure” will be experienced as negative or unpleasant (its valence rating is 1.70, with “1” anchoring the negative/unpleasant end of the scale). Some “failures” will be mild, some moderate, and some intense, and thus will be, on average, somewhat arousing (its arousal rating is 4.95). Kiverstein and Miller (2015) suggest that valence and arousal tap into basic and specific dimensions of emotion knowledge that can be abstracted across different situated contexts. Further, these are two important emotionality dimensions that are easily accessible to internal emotion systems evolutionarily designed to track basic and salient environmental features that may impact survival. For example, the automatic vigilance hypothesis (Pratto and John, 1991; Wentura et al., 2000) has been proposed to account for the attention-grabbing effects of negative and arousing stimuli, such that individuals who paid greater attention to negative and arousing stimuli were more likely to survive in past environments. Another hypothesis that has been proposed to account for how emotionally charged stimuli, regardless of valence polarity, may increase attention to environmental stimuli is the motivated attention and affective states hypothesis (Lang et al., 1990, 1997). Yet, as important as valence and arousal are in providing emotion information for conceptual knowledge, the results of the present study demonstrate that they do not exhaust all the potential dimensions of emotionality available through lived experience.

In the Introduction section we noted that Siakaluk et al. (2014) proposed that the emotional experience dimension taps into core person-environment regularities that exist across situated bodily contexts in which a given concept is relevant. The notion of situated conceptualization underscores the idea that although any particular situated context may have unique features (e.g., specific individuals, events, outcomes), aggregated across all situated contexts in which a given concept is relevant, there will likely be core person-environment regularities. We propose expanding what emotional experience may tap into in the following way. Kiverstein and Miller (2015) specifically emphasized that there are tight feedback links between cognition and emotion in conceptual processing, and as such the processing of emotion information is strongly associated with cognitive processes, such as reasoning, attention, memory, and planning. They stated that much neuroscience research suggests that there are “reciprocal interconnection[s] between cortical and subcortical structures” (p. 3), which allow for cognitive processes to influence affective processes *and* for affective processes to influence cognitive processes. With this in mind, we propose that core person-environment regularities across situated contexts that the dimension of emotional experience may track ought to be emotionally *and* cognitively salient or relevant to an individual; that is, that the regularities can be monitored by neural systems dedicated to the processing of perceptual, motor, emotion, and other forms of introspective information (such as interpretative and causal processing).

We agree with Kiverstein and Miller's (2015) suggestion that valence and arousal are basic affective processes that interconnect with cognitive processes such that the emotion information provided by these two dimensions of emotion experience “inform the organism that there is something in the environment of potential relevance or value” (p. 6). However, important these emotion-cognition interconnections are (and they are important), we suggest that there could be *higher-order* emotion-cognition interconnections that augment conceptual processing, and that this may be what the dimension of emotional experience measures. Before attempting to explicate this idea with the “failure” example below, it is important to underscore the idea that the more basic emotionality dimensions of valence and arousal and the potentially higher order dimension of emotional experience are also interconnected, such that there is likely to be overlap in what they are gauging across situated contexts. The correlational results of the present study support this conjecture, with correlations between emotional experience and extremity of valence ranging from 0.49 to 0.66, and correlations between emotional experience and arousal ranging from 0.37 to 0.57. Despite these relationships, our results suggest that emotional experience also measures something unique from either valence or arousal.

Returning to the “failure” example, at the heart of this concept is the understanding that a desired or expected outcome did not come about. What sorts of higher order emotion-cognition interconnections could the dimension of emotional experience be tracking across situated contexts, such as those listed above (sporting events, academic efforts, business endeavors, personal relationships, etc.)? Some possibilities include the following: manner of speaking with fellow agents (e.g., the tone of voice may change); retrieval from memory of past failures and accompanying affective states (e.g., thinking that the course of events cannot be changed and will therefore lead to an undesired outcome, and associated feelings of helplessness); focused attention on negative stimuli and not on positive stimuli (e.g., focusing on others who appear to share one's view that the situation is hopeless while ignoring those who are more positive); developing cognitive appraisals as to potential causes of the failure (e.g., noting lack of preparation, skill, or motivation); and contextually appropriate introspective inferences and judgments (e.g., updating one's self-esteem); among many others. To reiterate, we propose that although the specifics will be unique to each individual situated context, there will be core person-environment regularities that may be abstracted across all situated contexts in which “failure” occurs. The point we are emphasizing here is that the dimension of emotional experience may be tapping into conceptual knowledge that relies on the development of relatively more complex emotion-cognition feedback loops that arise through lived bodily experience across a wide array of different situated contexts.

Two other examples are potentially informative. First, consider the concept “success.” “Success” and “failure” differ greatly on the dimension of valence (their respective valence ratings are 8.29 and 1.70), but they happen to have precisely the same rating on the dimension of emotional experience (6.07). An intriguing question, therefore, is how qualitatively different types of experience (e.g., having a successful vs. unsuccessful personal relationship) may lead to similar emotional experience ratings. There are several possible components that may account for this. First, any major differences in the type of experience are likely to be accounted for by the dimensions of valence and arousal, which track more basic aspects of situated contexts, such as whether they are pleasant-unpleasant, and of low or high arousal. Second, although initially somewhat counterintuitive, the concepts of “success” and “failure” are similar in the sense that these concepts are embedded in many situated contexts that are rich in salient higher order emotion-cognition interconnections. The examples of higher order emotion-cognition interconnections provided above (manner of speaking with fellow agents; retrieval from memory of similar events and their outcomes and accompanying affective states; focused attention on some stimuli while ignoring other stimuli; developing cognitive appraisals as to potential causes; and contextually appropriate introspective inferences and judgments) suggest that similarly rich sets of core person-environment regularities may underlie concepts as different in valence as “success” and “failure.”

Further, the differences between “success” and “failure” are likely to be attenuated in the LDT. The development of both concepts will lead to rich sets of emotional-semantic features (what we've been referring to as core person-environment regularities) that may be brought to bear in the LDT. Although the set of core person-environment regularities associated with “success” is likely to be very different from the set associated with “failure,” the point here is that each of these two concepts will be associated with rich sets of emotional-semantic features. The activation of these sets of features will facilitate processing in the LDT for each concept, because their relative richness provides strong evidence that they are words (Pexman, 2012). Under different task demands, particularly those that require fine-grained decisions about meaning (e.g., using the criteria of “is the concept pleasant?” or “is the concept unpleasant?”), the differences between these two concepts may be more apparent.

Finally, consider the concept “cause,” for which the mean emotional experience rating is just 2.30 (its valence and arousal ratings are 5.07 and 4.20, respectively). Given this low emotional experience rating, we infer that it is unlikely that “cause” has a rich set of core person-environmental regularities that, crucially, are emotionally or cognitively salient or relevant to an individual. Instead, it may be that what is an underlying “cause” is interpreted or understood with a different concept; such as “hunger” to understand why someone ate something, or “anger” to understand why someone said something negative. In the LDT, the relatively impoverished set of emotional-semantic features associated with “cause” will not provide much evidence that it is a word, thus not facilitating LDT performance to the same extent that is the case for the concepts “success” and “failure.”

Of course, our account here is speculative. Certainly, these ideas will need to be tested in future research. Nonetheless, the present study demonstrates that semantic richness effects of emotional experience—faster latencies to words rated higher on this dimension—are observed in LDT. These effects were observed in three LDTs using different stimulus sets and suggest generalization to both nouns and verbs. These effects are independent of any effects of valence and arousal. These findings are important because they underscore the idea that knowledge gained through emotion experience is multifaceted. Intuition and reflection amply demonstrate that the dimensions of valence and arousal are important aspects of lived experience, and research cited above has demonstrated that these types of emotion knowledge influence performance in LDT. However, the results of the present study suggest that valence and arousal do not exhaust all dimensions of emotionality that underlie conceptual knowledge. The relatively new emotionality dimension of emotional experience accounts for LDT latency variability above and beyond that accounted for by valence and arousal. Taken together with previous research reporting effects of emotional experience in other visual word recognition tasks, it seems reasonable to conclude that emotional experience should be considered alongside such dimensions as valence and arousal in order to capture the multidimensionality of lexical (emotion) semantics.

## Author contributions

PS was involved in the planning of the study; analyses of the data for all three experiments; and primary writer of the manuscript. PN was involved in the planning of the study; collection and analyses of the data for Experiments 1 and 2; and the writing of the manuscript. BD was involved in the planning of the study; collection and analyses of the data for Experiments 1 and 2; and the writing of the manuscript. EL was involved in the analysis of the data for Experiment 3; and the writing of the manuscript. DS was involved in the collection of the data for Experiment 3; and the writing of the manuscript. MY was involved in the analysis and write up of the response latency distribution analyses of Experiment 1. PP was involved in the planning of the study; the collection of data for Experiment 3; and the writing of the manuscript.

## Funding

This research was supported by the Natural Sciences and Engineering Research Council (NSERC) of Canada in the form of Discovery Grants to PS and PP, an NSERC Alexander Graham Bell CGS M Scholarship to PN, and an NSERC Alexander Graham Bell CGS D to DS.

### Conflict of interest statement

The authors declare that the research was conducted in the absence of any commercial or financial relationships that could be construed as a potential conflict of interest.

## Footnotes

^1^There were two neutral words (*ether* and *rabbi*) with error rates greater than 30%, and they were removed from all descriptive and inferential analyses reported for the experiment.

^2^Discussion regarding the hierarchical multiple regression analyses for each experiment of the present study will focus on results at steps 2 and 3.

^3^Data for six stimuli were removed due to error rates in excess of 30%, and they were removed from all descriptive and inferential analyses reported for the experiment.
