# Supplementary material for: Effects of Emotional Experience in Lexical Decision
Source: Front Psychol. 2016 Aug 9;7:1157. doi: 10.3389/fpsyg.2016.01157 (PMC4977304; doi:10.3389/fpsyg.2016.01157)
Supplement: Supplementary file 1 [file DataSheet1.docx]

Appendix

Instructions Used to Collect Emotional Experience Ratings

Words differ in the extent to which they elicit or evoke an emotional experience. Some words elicit or evoke strong emotional experiences (e.g., JUSTICE), whereas other words elicit or evoke weaker emotional experiences (e.g., MOMENT). The purpose of this experiment is to rate words as to the ease with which they elicit or evoke emotional experience. For example, the word “justice” refers to a concept that is associated with high levels of emotional experience (e.g., think of the emotional conditions that arise when a jury verdict is delivered, such as joy, dismay, anger, frustration), whereas the word “moment” refers to a concept that is associated with low levels of emotional experience (i.e., it is difficult to think of any kind of emotional experience to which this word is related). Any word (e.g., “justice”) that in your estimation elicits or evokes high levels of emotional experience should be given a high emotional experience rating (at the upper end of the numerical scale). Any word (e.g., “moment”) that in your estimation elicits or evokes low levels of emotional experience should be given a low emotional experience rating (at the lower end of the scale). Because words tend to make you think of other words as associates, it is important that your ratings *not* be based on this and that you judge only the ease with which a word elicits or evokes emotional experience.

Your emotional experience ratings will be made on a 1 to 7 scale. A value of 1 will indicate a low emotional experience rating, and a value of 7 will indicate a high emotional experience rating. Values of 2 to 6 will indicate intermediate ratings. Please feel free to use the whole range of values provided when making your ratings. Type the number that is most appropriate for each word using the number pad on the computer keyboard. When making your ratings, try to be as accurate as possible, but do not spend too much time on any one word.

1 2 3 4 5 6 7

Low Medium High
